# Combining single-cell and bulk RNA sequencing to identify CAF-related signature for prognostic prediction and treatment response in patients with melanoma

**DOI:** 10.1038/s41598-025-14979-w

**Published:** 2025-08-08

**Authors:** Jing Wang, Ying Song, Zifu Li, Tianxiang Gao, Wei Shen, Zijian Kang, Chong Xu

**Affiliations:** 1 Shanghai Center for Clinical Laboratory, Shanghai, China; 2 Neurovascular Center, Changhai Hospital, Shanghai, China; 3 Department of Laboratory Medicine, Renji Hospital, School of Medicine, Shanghai Jiao Tong University, Shanghai, China; 4 Department of Rheumatology and Immunology, Shanghai Sixth People’s Hospital, Shanghai Jiao Tong University School of Medicine, Shanghai, China

**Keywords:** Cancer-associated fibroblasts, Melanoma, Single-cell RNA sequencing, Prognostic biomarker, Risk factors, Cancer, Skin cancer, Tumour biomarkers

## Abstract

Cancer-associated fibroblasts (CAFs) play complex roles in the tumor microenvironment (TME) of melanoma. However, their impact on prognosis and treatment response in melanoma remains unclear. In this study, ScRNA-seq data (GSE115978) were utilized to characterize CAF heterogeneity and identify marker genes in melanoma. Prognostic CAF genes were identified from the TCGA dataset and employed to construct a risk signature, which was subsequently validated in an independent cohort (GSE65904). Mutation, copy number variation (CNV), pathway enrichment, immune infiltration, and drug sensitivity were analyzed to determine the signature’s clinical relevance. Immunohistochemistry (IHC), immunofluorescence (IF), and qPCR were performed to validate the expression of CAF signatures on clinical melanoma samples. We identified CAFs in patients with melanoma through single-cell RNA sequencing data. A 28-gene CAF signature was constructed using the Least Absolute Shrinkage and Selection Operator (LASSO) regression based on 271 prognostic CAF genes. This signature demonstrated excellent prediction accuracy for survival, with area under the curve (AUC) values of 0.737, 0.737, and 0.779 for 1-year, 3-year, and 5-year survival, respectively. The signature was an independent prognostic factor and was correlated with CNVs, and immunosuppressive TME features (reduced CD8^+^ T cells, M1 macrophages). Additionally, our CAF signature could predict the efficacy of multiple chemotherapy drugs and serve as a potential prognostic marker for immunotherapy. Experimental validation confirmed the expression of CAF signature genes in melanoma tissue. Our model may help predict the prognosis and response to chemotherapy and immunotherapy in patients diagnosed with melanoma.

**Supplementary Information:**

The online version contains supplementary material available at 10.1038/s41598-025-14979-w.

## Introduction

The tumor microenvironment (TME) is a complex ecosystem composed of malignant and stromal cells, such as fibroblasts. Cancer-associated fibroblasts (CAFs) are one of the most abundant stromal cell types found in almost all solid tumors^1^. Extensive research has indicated that CAFs play crucial roles in promoting tumor development through multiple mechanisms, such as tumor invasion, maintenance of cancer stemness, resistance to chemotherapy, and evasion of immune surveillance^2,3^. However, it should be noted that in certain cancer types, studies have shown that CAFs can exert tumor-suppressive effects within the TME^4,5^. Therefore, it is essential to ensure precise targeting of pro-tumorigenic CAFs, better characterization of CAF in prognosis, and immunotherapy response of tumors.

Melanoma is an aggressive skin cancer with limited treatment options in advanced stages^6,7^. The cross-talk between CAFs and melanoma cells has been reported to promote melanoma progression and metastasis^8,9^. Bulk transcriptomic analyses have identified several CAF-related gene signatures associated with poor prognosis in various cancers^10,11^. However, the specific roles of CAF-related genes in melanoma, particularly their impact on prognosis and immunotherapeutic response, remain unclear. Furthermore, bulk sequencing techniques average gene expression across all cell types, thus obscuring cell type-specific expression patterns. Recent advances in single-cell RNA-sequencing (scRNA-seq) have enabled high-resolution dissection of cell heterogeneity and signaling dynamics between cell subsets within complex tissues^12^. Combining scRNA-seq and bulk RNA-seq data allows the identification of cell type-specific signatures while retaining statistical power for clinical outcome association analyses^13,14^.

This study aimed to leverage scRNA-seq data to identify CAF-related genes and construct a gene signature specifically related to CAFs in melanoma. Our objective was to investigate the correlation between this CAF signature and clinical outcomes in patients and gain insights into the molecular mechanisms and drug responses associated with this CAF signature in melanoma. The findings of this study may shed light on the molecular interplay between CAFs and melanoma cells and on prognostic biomarkers and therapeutic targets.

## Methods

### Data resource

The scRNA-seq data were obtained from the dataset GSE115978^15^, which contained samples from 16 patients with malignant melanoma. RNA sequencing and clinical data of melanoma were downloaded from the UCSC Xena database (http://xena.ucsc.edu/) and used as training datasets. The validation dataset GSE65904^16^ was downloaded from the GEO database (http://www.ncbi.nlm.nih.gov/geo/) for further validation. Datasets related to PD-1 immunotherapy in patients with melanoma were obtained from the Gene Expression Omnibus (GEO) database under accession numbers GSE78220^17^ and GSE145996^18^. Supplement Tables 1 and 2 show the demographic characteristics for the training and validation sets.

### ScRNA-seq analysis

The Seurat package was used to possess the raw data from this study, performed quality control, unbiased clustering, and ran uniform manifold approximation and projection (UMAP) nonlinear dimensional reduction. The NormalizeData function was used to normalize the unique molecular identifier counts. The FindVariableGenes function identified the top 1000 highly variable genes. Principal component analysis was performed on the variable genes using the RunPCA function. The FindIntegrationAnchors and IntegrateData functions were then used to correct the batch effects for each patient. Cell clustering analysis was performed using a K-nearest neighbor graph. UMAP plots visualized the clustering information, and cell types were annotated based on known cellular marker expression patterns.

### Functional analysis of CAF-related genes

To explore the functional roles of identified CAF-related genes, we performed gene ontology (GO) and Kyoto Encyclopedia of Genes and Genomes (KEGG) pathway enrichment analysis using the R package ClusterProfiler^19^. Overrepresented GO terms and KEGG pathways were identified based on a hypergeometric test with false discovery rate (FDR)-adjusted p-values < 0.05, which were considered statistically significant. The enriched terms were visualized as dot plots generated using the dotplot function, where the size of the dot represents the gene ratio (the ratio of identified genes to total genes in that term), and the color represents the adjusted p-value.

### Prognostic analysis of CAF-related genes

To evaluate the prognostic value of the identified CAF marker genes, melanoma patients in the discovery dataset were stratified into high- and low-expression groups based on the median CAF signature score. Patients with scores equal to or above the median were assigned to the high-expression group, while those with scores below the median were assigned to the low-expression group. This cutoff value was applied to the validation dataset to test accuracy. Kaplan-Meier survival analysis using the R packages survival and survminer assessed differences in overall survival between the high and low expression groups. The log-rank test determined the statistical significance of survival differences. CAF marker genes with log-rank *p* < 0.05 were considered to have a significant prognostic value. Hazard ratios (HR) and 95% confidence intervals (CI) were calculated via univariate Cox proportional hazards regression to quantify the correlation between gene expression and patient survival.

### Construction of CAF-related signature

Univariate Cox regression models were constructed with the CAF subgroup marker genes using survival time, survival status, and the Coxph function in the R survival package. Genes significantly associated with prognosis (*p* < 0.05) were selected as prognosis-related CAF genes. To construct a parsimonious prognostic model, least absolute shrinkage and selection operator (LASSO) regression was applied using the glmnet package: CAF genes were subjected to 10-fold cross-validation to select the optimal penalty parameter (λ) that minimized partial likelihood deviance, and non-zero coefficient genes at λmin were used to form the final CAF-related signature. The risk score was calculated as the weighted sum of gene expression (Risk Score = ∑Coefficienti×Expression). The training cohort was stratified into high- and low-risk groups using the median risk score. Time-dependent ROC curves were generated via the timeROC package to evaluate predictive accuracy at 1/3/5 years, while Kaplan-Meier survival analysis with log-rank test was performed using survminer to assess differences between risk groups. Clinical associations were visualized using ggplot2, comparing risk groups across categorical variables via chi-square tests and continuous variables via Wilcoxon tests.

### Evaluation of CAF-related signature as an independent prognostic factor

Univariate and multivariate Cox analyses were performed using the Coxph function in R on the clinical data from the training and test datasets to evaluate whether the CAF-related signature was an independent, significant prognostic factor. A p-value threshold 0.05 determined significance in both the univariate and multivariate analyses.

### Mutation analysis of the CAF-related signature

The R package maftools compared mutation levels between high- and low-risk groups classified by the CAF-related signature. Fisher’s exact test detected if any of the top 20 mutated genes had significantly different mutation rates between the two risk groups (*p* < 0.05).

### Copy number variation analysis of the CAF related signature

Copy number variation (CNV) data were processed using the GISTIC 2.0 tool within GenePattern with default settings^20^. GISTIC 2.0 identifies regions of focal amplification and deletion by evaluating the frequency and amplitude of copy number changes across multiple samples. Regions were defined as significant based on a q-value cutoff of 0.25. The R package ggplot2 was used to plot the distribution of CNV regions across the genome for the CAF-related signatures.

### Hallmark pathway enrichment difference analysis of the CAF-related signature

Hallmark gene sets and TCGA expression data were input into the ssgsea method in the GSVA R package to estimate pathway enrichment scores for each sample. The pheatmap R package visualized the differences in enrichment scores between the risk groups across hallmark pathways. T-tests analyzed statistical the differences in each pathway between groups.

### Analysis of immune characteristics by CAF-Related signature

The R package estimate was used to assess the immune, stromal, and estimate scores for each sample based on the expression profiles. The ggplot2 package was then used to plot differences in these scores between the high- and low-risk groups defined by the signature, and t-tests were used to test for the significance of differences between them.

### CAF-related signature immune infiltration analysis

Immune cell proportions were estimated from bulk tumor gene expression profiles using the CIBERSORT, XCELL, and EPIC algorithms implemented in the R package IOBR. Violin plots were generated using the ggplot2 package in R to visualize the differences in immune cell proportions between the high and low CAF-related signature groups. The statistical significance of the differences in immune cell proportions was determined using two-sided t test.

### CAF-related signature chemotherapy resistance analysis

The pRRophetic R package predicted drug sensitivity by estimating the IC50 values from the Genomics of Drug Sensitivity in Cancer (GDSC) database. Pearson’s correlation was used to compare the CAF-related signature scores with the IC50 values for each drug. Drugs with significantly different area under the curve (AUC) between the high and low CAF-related signature groups (*P* < 0.05) were significantly associated with CAF-related chemotherapy resistance.

### CAF-related signature immunotherapy prognosis analysis

To evaluate the prognostic relevance of the CAF-related signature in immunotherapy, we analyzed the IMvigor210 cohort of patients with carcinoma who were treated with anti-PD-L1 therapy. Risk scores were calculated based on the CAF signature, and patients were stratified into high- and low-risk groups according to the median value. Survival differences were assessed using Kaplan-Meier analysis and the log-rank test. CAF scores were compared across clinical response categories (PD, CR, PR, SD) using the Wilcoxon rank-sum test. The predictive accuracy of the signature was evaluated by receiver operating characteristic (ROC) curve analysis with AUC calculation. To validate these findings, we performed Gene Set Enrichment Analysis (GSEA) in two independent anti-PD-1 melanoma cohorts, GSE78220 and GSE145996, to assess CAF signature enrichment in responders versus non-responders. Statistical analyses were conducted using R (v4.2.1), with significance defined as *P* < 0.05.

### Clinical sample collection and tissue handling

We collected 15 fresh-frozen melanoma specimens and paraffin-embedded tissues from Shanghai Renji Hospital. All patients signed informed consent forms, and the study was approved by the hospital’s ethics committee (Ethics approval No. KY2024-066-C). The collected tissue samples were immediately fixed in 4% paraformaldehyde solution for 24 h and then embedded in paraffin for subsequent immunohistochemical and immunofluorescent staining analyses. Meanwhile, part of the tissue samples was quickly frozen in liquid nitrogen and stored in a -80℃ refrigerator for total RNA extraction and quantitative polymerase chain reaction (qPCR) analysis.

### Immunohistochemical staining

Immunohistochemical (IHC) staining was used to detect the protein expression levels of CAF-related genes in the tumor tissues. First, paraffin-embedded tissue sections were placed on slides, and dewaxing, hydration, and antigen retrieval were performed. Then, the sections were incubated with 3% H₂O₂ solution at room temperature for 15 min to inactivate endogenous peroxidase activity. Next, the sections were incubated with primary antibodies (CAF-related genes, diluted according to the manufacturer’s instructions) in a humidified box at 4℃ overnight. The next day, after washing the sections with PBS, they were incubated with corresponding secondary antibodies at room temperature for 30 min. Finally, a DAB chromogenic kit was used for the chromogenic reaction, and the nuclei were counterstained with hematoxylin.

### Immunofluorescent staining

Paraffin sections were dewaxed and hydrated for immunofluorescence (IF) staining, followed by antigen retrieval. Then, the sections were treated with 0.3% Triton X-100 for 10 min to enhance antibody permeability. Primary antibodies (COL1A2, MFSD5, NOTCH3) were applied and incubated at 4℃overnight. After washing with PBS, the sections were incubated with corresponding fluorescence-labeled secondary antibodies at room temperature in the dark for 1 h. The nuclei were stained with DAPI, and the sections were mounted. Images were observed and captured under a fluorescence microscope, and co-localization analysis was performed to evaluate the spatial relationship between CAF-related genes.

### Total RNA extraction and cDNA synthesis and qPCR detection

Total RNA was extracted from the frozen tissue samples using TRIzol reagent, according to the manufacturer’s instructions. The concentration and purity of the extracted RNA were determined by a Nanodrop spectrophotometer. Then, mRNA was reverse-transcribed into cDNA using the PrimeScript RT Master Mix reverse transcription kit, following the kit’s instructions. qPCR was used to measure the mRNA expression levels of CAF-related genes in tumor and normal tissues. With cDNA as the template, the reaction was performed using SYBR Premix Ex Taq II qPCR reagent with the following steps: 30 s of pre-denaturation at 95℃, followed by 40 cycles of 5 s of denaturation at 95℃ and 30 s of annealing and extension at 60℃. Each sample was run in triplicate, and β-actin was amplified simultaneously for data normalization. The relative expression levels of target genes were calculated using the 2⁻^ΔΔ^Ct method, where ^ΔΔ^Ct = (Ct value of the target gene in tumor tissue - Ct value of β-actin in tumor tissue) - (Ct value of the target gene in normal tissue - Ct value of β-actin in normal tissue).

## Results

### Identification of CAF marker genes in the melanoma

**Fig. 1:**
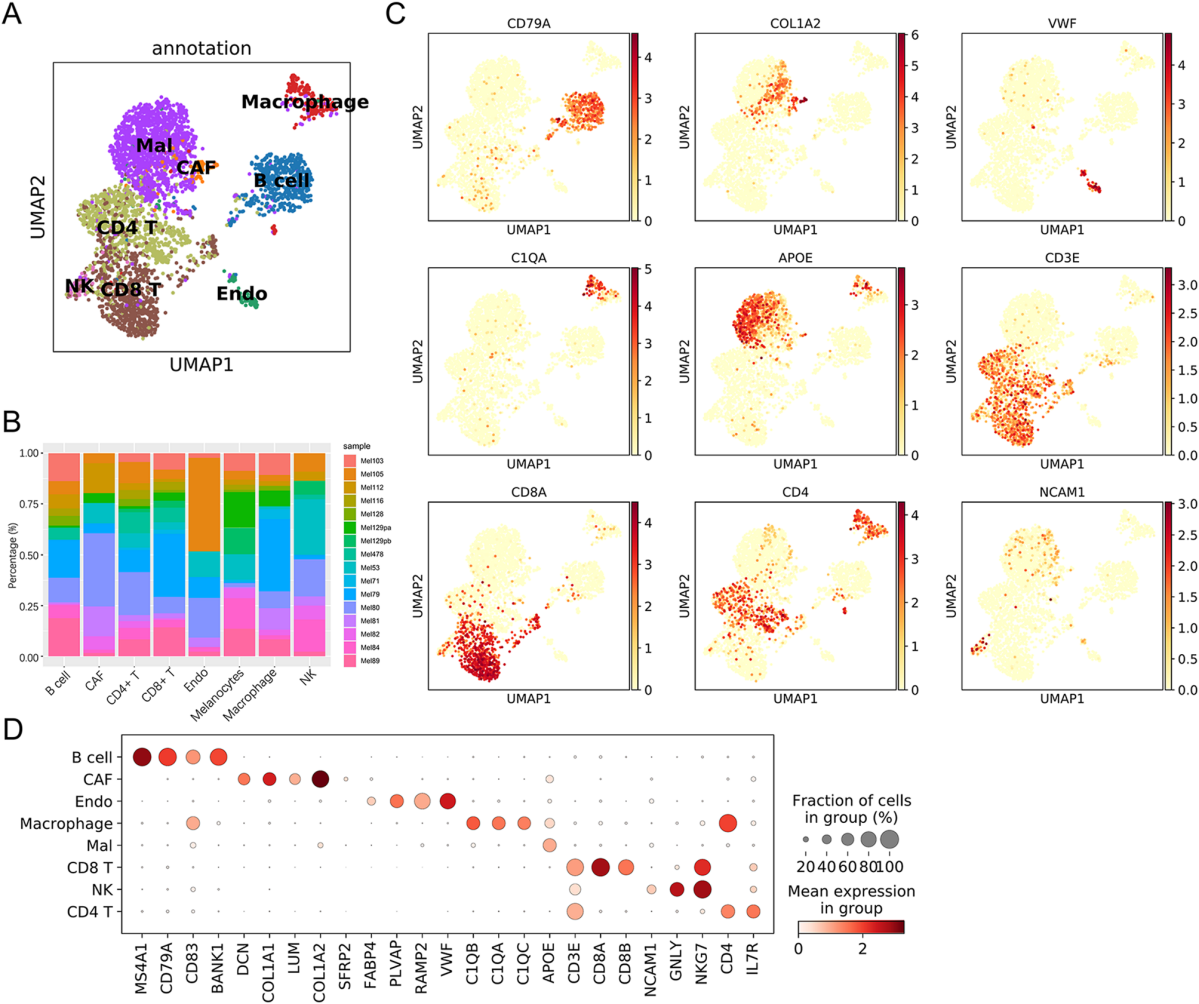
Identification of melanoma cell subsets. (**A**) UMAP showing the cell clusters in melanoma patients. (**B**) Bar plots of the cellular sources for 8 clusters in melanoma patients. Blocks represent different samples, and block heights represent the proportion of detected cells. (**C**) Heatmaps showing the marker genes of different cell clusters. (**D**) Dot plot showing the marker genes of different cell clusters. The dot color represents the gene expression level; size represents the proportion of positive cells.

To identify melanoma cell types, we obtained public scRNA sequencing data from the GEO dataset GSE115978. We obtained 3,630 high-quality single-cell data after quality control processing and then performed normalization, unsupervised dimensionality reduction, graph-based clustering, and cell type identification on these data (Fig. 1A). Figure 1B shows the proportion of individuals across different cell types. The proportion of each cell type was spread across all individuals, without any sample showing a unique or skewed profile. This divergent, interleaved distribution demonstrates that variations in cell type abundance are driven by biological heterogeneity rather than by technical batches. The cell types were annotated based on canonical known markers, such as CD3D for T cells, CD79A for B cells, C1QA for macrophages, and VWF for endothelial cells (Fig. 1C). We also calculated the marker gene expression for each cell type (Fig. 1D). A total of 1260 marker genes for CAFs were identified based on a criterion of adjusted p-value < 0.05 and fold change > 1, including DCN, COL1A1, LUM, and COL1A2 (Fig. 1D). To characterize the functional relevance of CAFs, we performed GO and KEGG enrichment analyses (Supplement Fig. 1A-D). GO enrichment showed that CAF marker genes were enriched for “extracellular matrix organization”, “actin and cadherin binding”, and “cell-cell junction” functions. KEGG pathway analysis revealed enrichment for “PI3K-Akt signaling”, “focal adhesion”, and “ECM-receptor interactions”. These results suggest that the primary function of CAFs is to secrete and remodel the extracellular matrix.

### Construction of CAF-related risk model in patients with melanoma

**Fig. 2:**
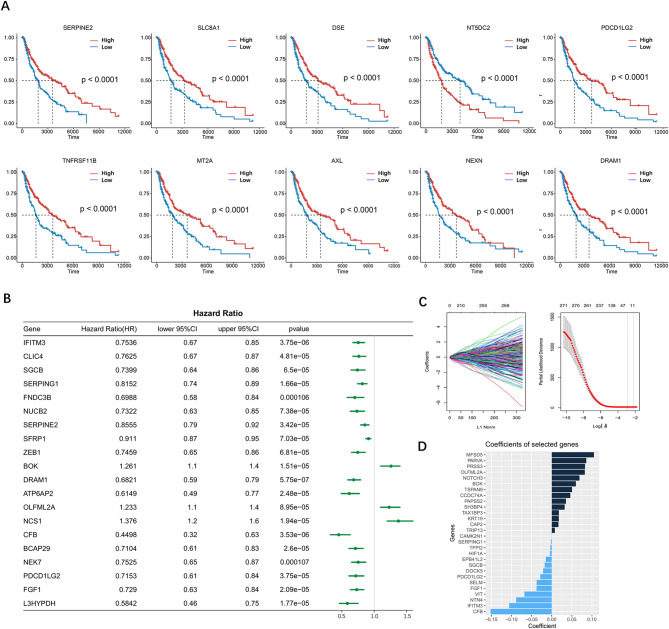
Construction of CAF-related signature in melanoma patients. (**A**) Kaplan-Meier survival curves of representative CAF marker genes with significant prognostic differences. (**B**) Forest plot showing the univariate Cox regression of prognosis-associated genes. (**C**) LASSO screening (left) and cross-validation (right) to select the optimal adjustment parameter. (D) Selected features and their coefficients in the CAF model.

To explore the impact of CAFs on the prognosis of melanoma patients, we divided patients into high- and low-expression groups based on the median expression of CAF marker genes and conducted survival analysis. Figure 2 A illustrates the Kaplan-Meier survival curves of the top 10 prognostic CAF marker genes. Most CAF marker genes exhibited a protective effect on survival, including SERPINE2, SLC8A1, DES, PDCD1LG2, TNFRSF11B, MT2A, AXL, NEXN, and DRAM1. Conversely, high NT5DC2 expression was correlated with poor survival prognosis. We then performed univariate Cox regression analysis and identified 271 CAF genes that were significantly associated with melanoma patient survival time. Among the top 20 ranked genes, BOK, OLFML2A, and NCS1 indicated worse survival, while the other genes demonstrated a favorable effect (Fig. 2B). We then used the LASSO regression model to extract 28 features from these genes and construct a prognostic prediction model, including MFSD5, PARVA, PRSS3 and NOTCH3 (Fig. 2C**)**. The risk score was calculated based on the expression level and risk coefficient of each gene (Fig. 2D, Supplement Table 3).

### Validation of CAF signature expression in melanoma tissues

**Fig. 3:**
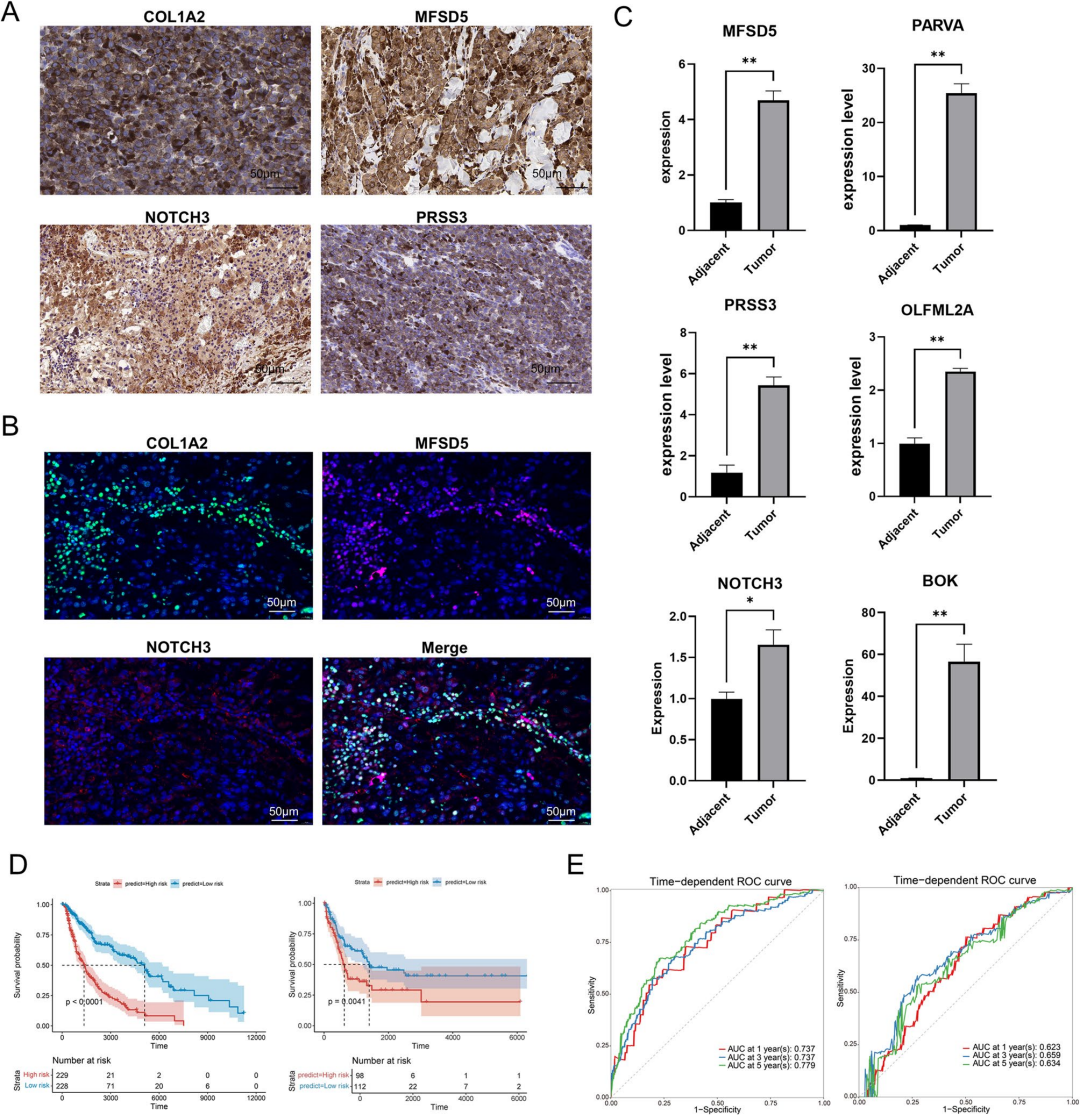
CAF related signature in the training dataset and the validation dataset. (**A**) Representative immunohistochemistry (IHC) images of COL1A2, MFSD5, NOTCH3, and PRSS3. Brown staining indicates positive expression of the respective proteins, with a scale bar of 50 μm showing the magnification level. (**B**) Immunofluorescence (IF) images of COL1A2, MFSD5, NOTCH3, and a merged image of these proteins. Different colors represent different proteins, and nuclei are blue, with a scale bar of 50 μm. (**C**) qPCR validating the expression levels of MFSD5, PARVA, PRSS3, OL FML2A, NOTCH3, and BOK between tumor and adjacent tissues. (**D**) Kaplan-Meier curves showing the survival state of patients with melanoma in the training dataset (left) and the validation dataset (right). (**E**) ROC curves for the 1, 3, 5-year survival prediction in the training dataset (left) and the validation dataset (right).

To further validate the expression patterns of the identified genes, we conducted IHC, IF and qPCR experiments. The IHC results demonstrated that CAF signature genes COL1A2, MFSD5, NOTCH3, and PRSS3 were highly expressed in tumor tissues (Fig. 3A). IF further revealed that these proteins were enriched within the tumor tissues, with the merged images highlighting their co-localization within the tumor microenvironment (Fig. 3B). The qPCR analysis quantitatively confirmed the upregulation of MFSD5, PARVA, PRSS3, OLFML2A, and NOTCH3 at the mRNA level in tumor tissues, with statistically significant differences compared to adjacent tissues (Fig. 3C). The above results confirm that the identified CAF signature genes are highly expressed in melanoma tumor tissues, underscoring their potential role in melanoma biology.

### Prognostic value of CAF-related risk model

To evaluate the prognostic prediction accuracy of the risk model, we incorporated an independent validation dataset comprising 214 melanoma patients (GSE65904). Patients with melanoma in both the training and validation datasets were divided into high- and low-risk score groups based on the cut-off value in the discovery set (Supplement Fig. 2A-D). Patients in the high-risk group exhibited a less favorable prognosis and shorter survival times, while low-risk patients had more positive outcomes in both datasets (Fig. 3D). CAF genes were also differentially expressed between the high- and low-risk groups (Supplement Fig. 3A-B). ROC curves indicated high predictive accuracy, with AUC values of 0.737, 0.737, and 0.779 for 1-year, 3-year, and 5-year survival rates, respectively, in the training set (Fig. 3E). In the validation set, the AUC values for 1-, 3-, and 5-year survival rates were 0.623, 0.659, and 0.634, respectively (Fig. 3E). Thus, the CAF-related risk score could predict the prognosis of patients with melanoma.

### Relationship between CAF-related risk score and clinical characteristics of melanoma patients

**Fig. 4:**
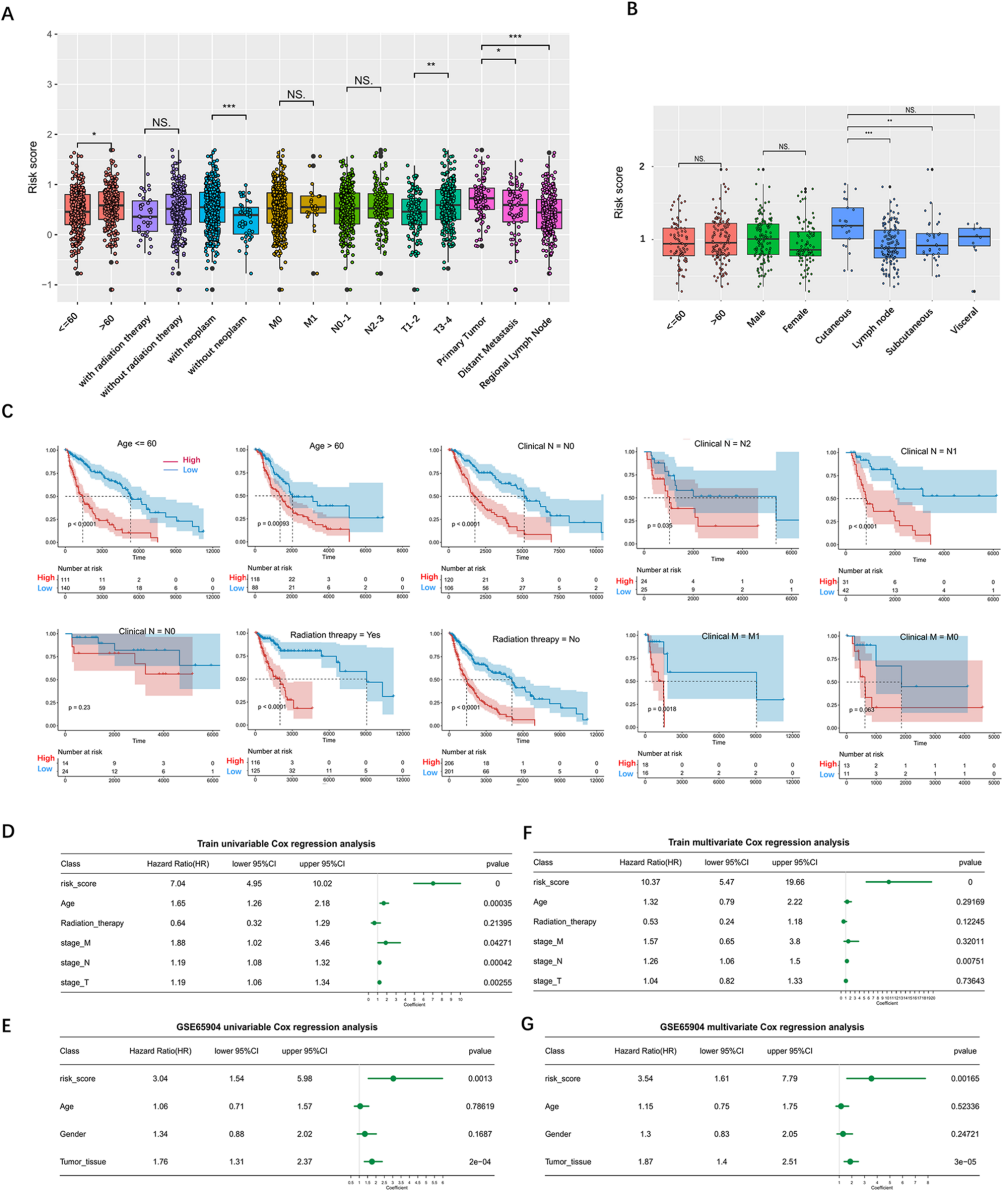
Correlation between CAF and clinical characteristics of melanoma patients. (**A**-**B**) Differences in CAF-related signature in clinical groupings of the training set (**A**) and validation data set (**B**). (**C**) Kaplan-Meier survival plots of high - and low-risk groups within different clinical groups. (**D**-**E**) Univariate Cox analysis of the training dataset (**D**) and the validation dataset (**E**). (**F**-**G**) Multivariate Cox analysis of the training dataset (**F**) and the validation dataset (**G**).

To elucidate the association between the CAF-related risk score and the clinical characteristics of patients with melanoma, we conducted a comparative analysis of risk scores among patients exhibiting diverse clinical features. The results revealed that tumor stage and location were correlated with risk scores in the training dataset (Fig. 4A**)**. Additionally, tumor location was correlated with the risk score in the validation dataset (Fig. 4B**)**. To assess the applicability of the model, we examined survival outcomes among different clinical cohorts and found significant survival discrepancies between the high- and low-risk groups across nearly all clinical cohorts (Fig. 4C**)**. To ascertain whether the CAF-related risk score acts as an independent prognostic factor, we performed univariate and multivariate Cox regression on the risk scores from both the training and validation datasets, incorporating various clinical characteristics (Fig. 4D-G). Notably, we observed that the risk score was not affected by factors such as age and sex and was an independent prognostic risk factor for melanoma in both datasets. These results demostrate the reliability of the CAF-related risk model.

### Relationship between CAF-related risk score and gene alteration

**Fig. 5:**
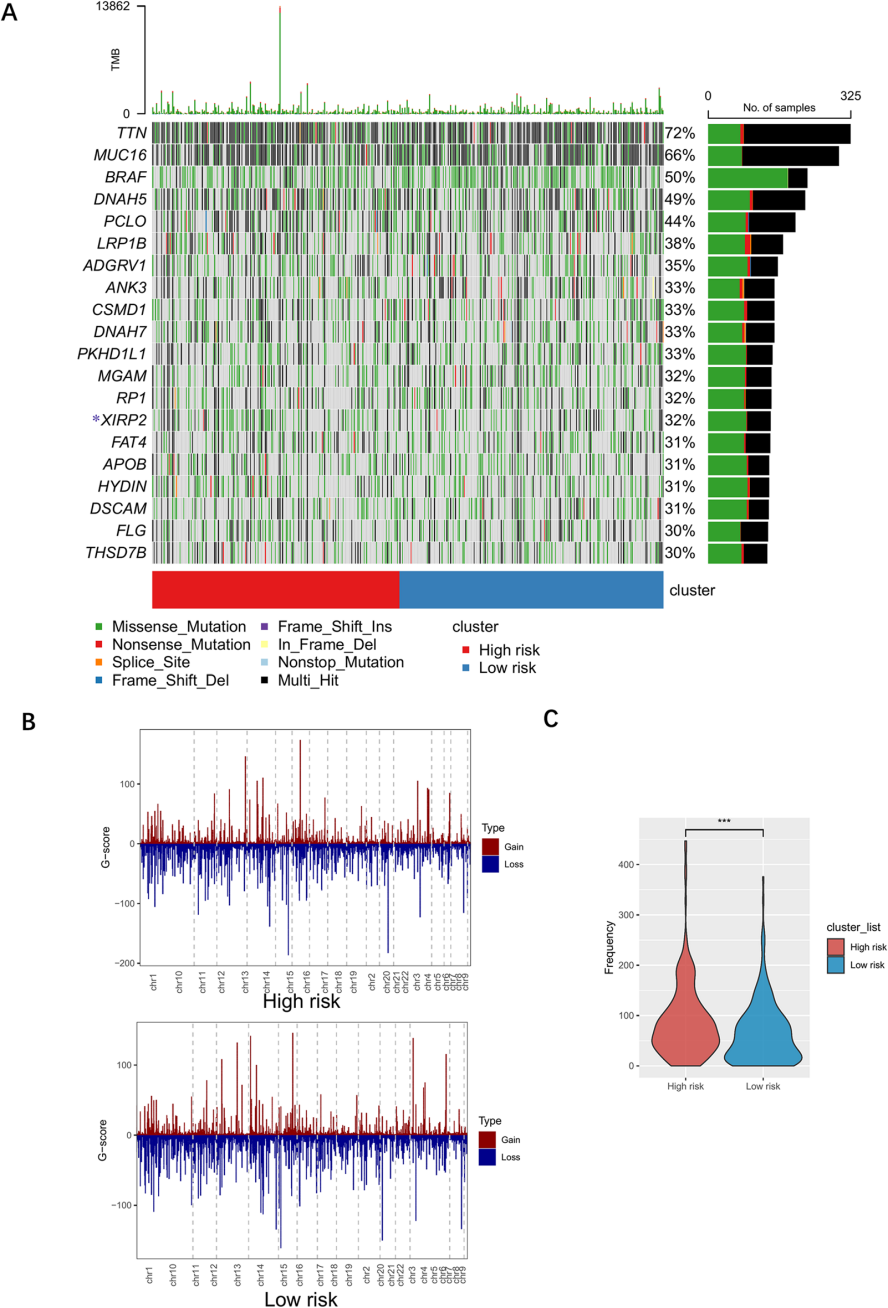
Gene mutations and copy number variations frequency between high and low risk groups of CAF-related signature. (**A**) CAF-related signature gene mutations between high and low risk groups. (**B**) Distribution of copy number variants in patients with high and low CAF-related signature. (**C**) Violin plot showing the frequency between high and low CAF-related signature groups.

To investigate the variations in mutation levels between the high- and low-risk CAF score groups, we conducted a comparative analysis of CAF-related signature mutations between these groups. Among the melanoma patients, the most frequently mutated genes were TTN, MUC16, BRAF, DNAH5, and PCLO (Fig. 5A**)**. Employing Fisher’s exact test on the top 20 mutated genes, we found that the established driver mutations, including BRAF and TTN, did not show significant associations​​ with the CAF-related risk score. In contrast, XIRP2, a cytoskeletal regulator linked to metastasis in other cancers, exhibited higher mutation rates in the high-risk group (*p* < 0.05).

We also examined the association between CNV frequency and the risk score (Fig. 5B**)**. Both groups exhibited copy number gains in chr8(p23.3) (ERICH1) and chr16(q12.2) (CES1P1), and copy number losses in chr1(p31.3) (PATJ) and chr15(q14) (ACTC1). CNVs in RAB31, INHBA, GLI3, and PPP4R1 were exclusively observed in the high-risk group. Statistical analysis revealed that the amplification frequency was significantly higher in the high- versus low-risk groups, indicating the correlation between risk score and CNVs in melanoma (Fig. 5C**)**.

### Differences in pathways and immune infiltration between High- and Low-Risk CAF related signature groups

**Fig. 6:**
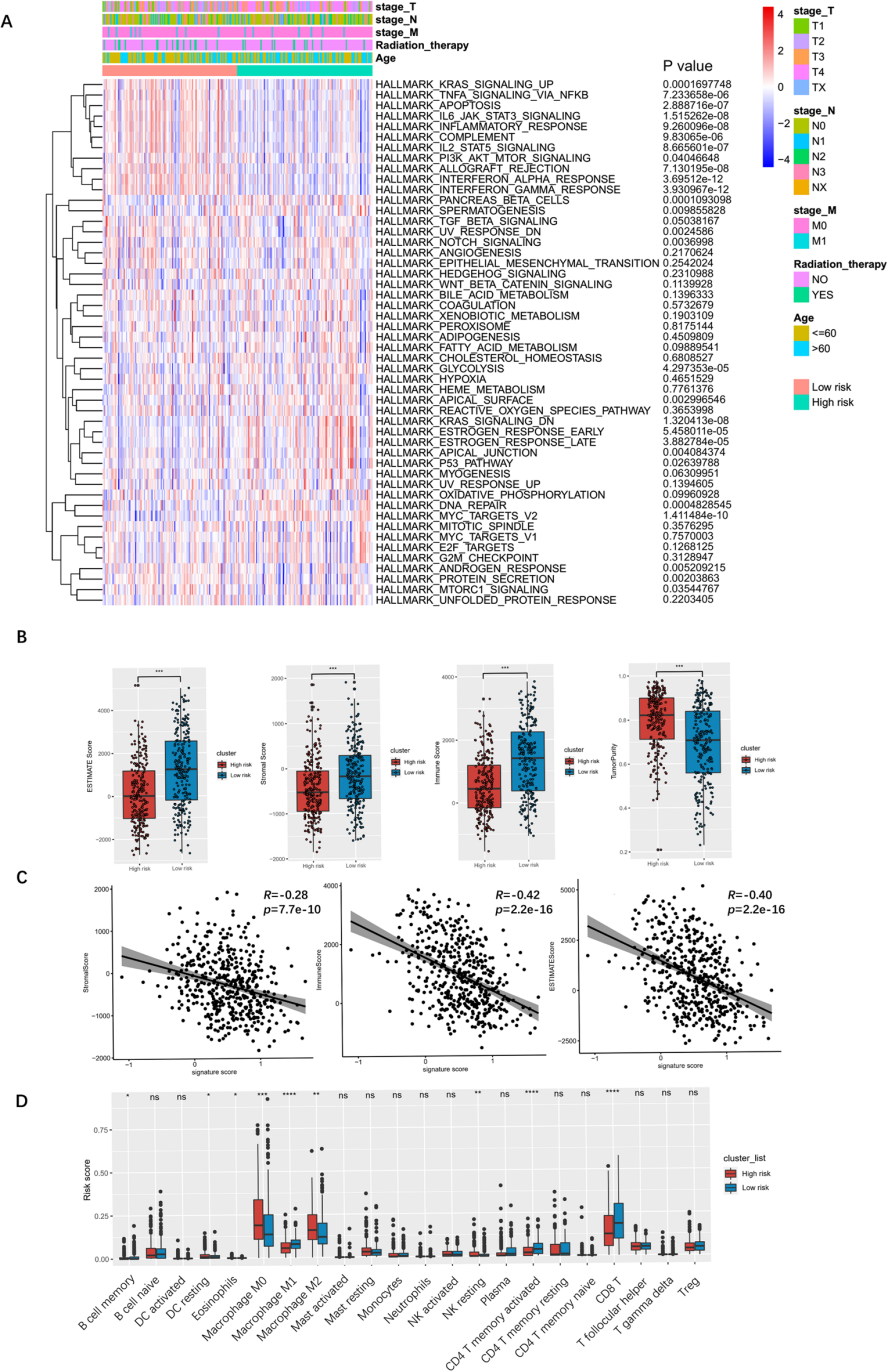
Differences in hallmark enrichment and immune characteristics among CAF-related signature groups. (**A**) Enriched hallmark pathways related to CAF groups. (**B**) Estimate the score difference between the high and low CAF groups. (**C**) Dot plots showing the correlation between CAF and estimate scores. (**D**) Boxplot showing the immune infiltration characteristics of tumor microenvironment between low and high-risk groups.

To evaluate pathway disparities between high- and low-risk CAF groups, we conducted gene set variation analysis (GSVA). The high-risk group exhibited downregulation of “KRAS signaling”, “Apoptosis”, and inflammatory response pathways (IL-6/JAK-STAT3, IL-2/STAT5, TNF-α/NF-κB), while showing upregulation of “DNA repair”, “p53 signaling”, and “Notch signaling” pathways (Fig. 6A**)**. These findings suggest that CAF scores are associated with immune activation and tumor-promoting mechanisms. Utilizing the ESTIMATE method, we found that the high-risk group demonstrated lower immune and stromal scores but higher tumor scores (Fig. 6B**)**. Correlation analysis confirmed that the CAF score was significantly negatively associated with immune scores (*R* = − 0.42, *p* < 0.001), ESTIMATE scores (*R* = − 0.40, *p* < 0.001), and stromal scores (*R* = − 0.28, *p* < 0.05) (Fig. 6C**)**. These negative correlations indicate an inverse relationship between the CAF score and immune/stromal components. To further investigate the TME, we used CIBERSORT to estimate immune cell proportions. High-risk patients exhibited higher proportions of pro-tumor immune cells (M0 macrophages, M2 macrophages, and dendritic cells) and lower proportions of anti-tumor immune cells (M1 macrophages, CD4^+^ memory T cells, and CD8^+^ T cells) compared to low-risk patients (Fig. 6D). The decreased M1 macrophages may downregulate inflammatory responses, while fewer CD8^+^ T cells could facilitate immune escape, thereby explaining the poorer prognosis in the high-risk group. The above results highlight the immunosuppressive and tumorigenic features of the high-risk CAF group.

### Association between CAF-Related signature and chemotherapy resistance

**Fig. 7:**
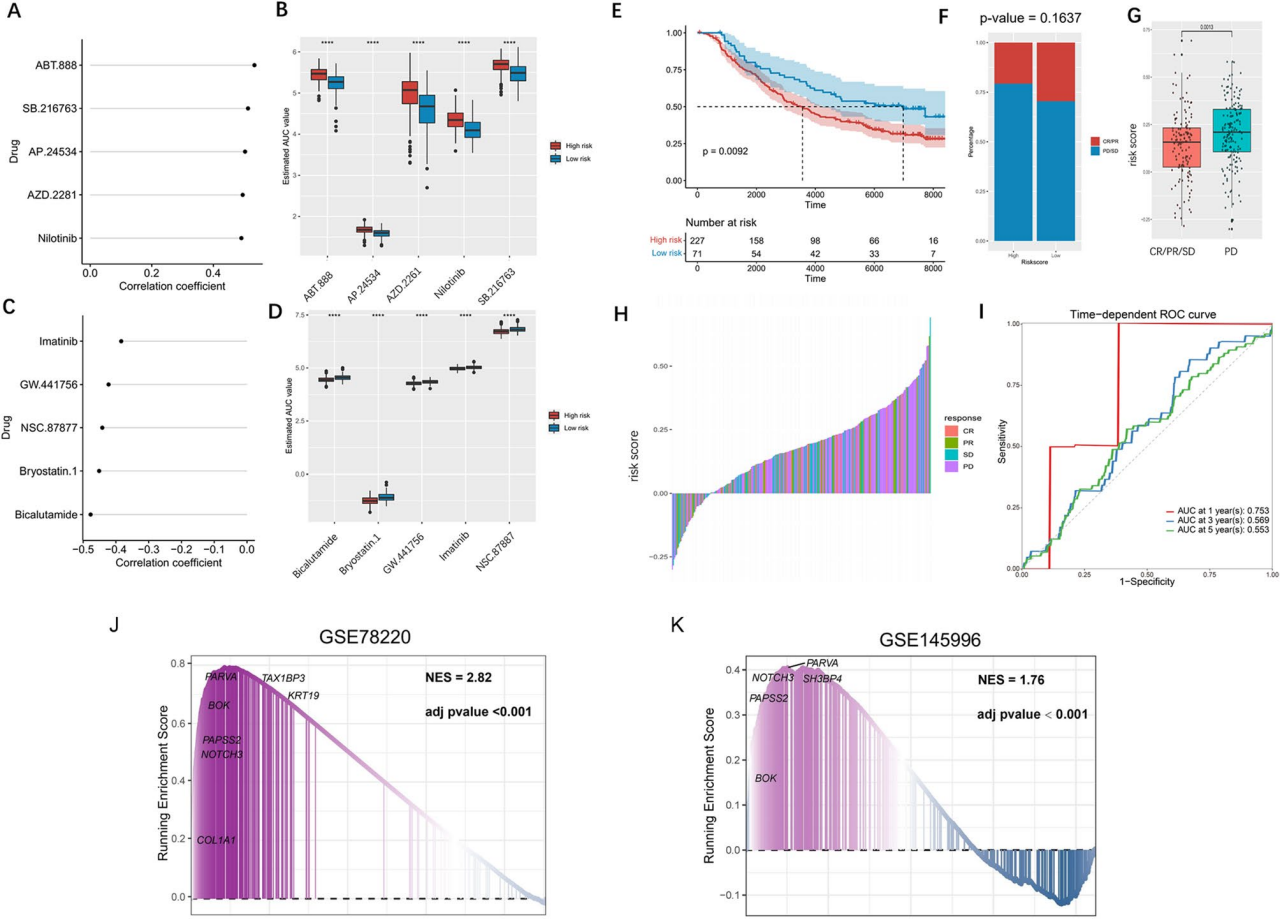
Association between CAF-related signature and chemoresistance. (**A**) IC50 values of the top five drugs which were significantly positively correlated with CAF-related signatures. (**B**) Distribution of AUC of five drugs between high and low-risk groups. (**C**) IC50 values of the top five drugs which were significantly negatively correlated with CAF-related signature. (**D**) Distribution of AUC of five drugs between high and low-risk groups. (**E**) Survival curves of the treatment cohort’s high - and low-risk groups. (**F**) The efficacy of immunotherapy between the high-risk and low-risk groups in the treatment cohort. (**G**) Risk scores between different immunization groups. (**H**) Risk scores distribution in the treatment cohort data. (**I**) ROC curve of the reliability of the immunotherapy response. (**J**-**K**) Gene set enrichment analysis (GSEA) results for CAF-signature in the GSE78220 and GSE145996 datasets.

To evaluate the association between the CAF-related signature and chemotherapy resistance, we applied the pRRophetic algorithm to predict drug sensitivity (IC50 values) using transcriptomic profiles from the GDSC. Figures 7A-D depict the top 5 drugs exhibiting significant positive and negative correlations with the CAF signature. Among the top drugs with significant correlations, imatinib (a PDGFR/ABL/KIT inhibitor) and bicalutamide (an androgen receptor antagonist) exhibited strong negative correlations with CAF risk scores, suggesting enhanced efficacy in high-risk patients compared to low-risk patients. Conversely, paclitaxel (a microtubule inhibitor) and vemurafenib (a BRAF inhibitor) showed positive correlations, indicating potential sensitivity in the low-risk group.

### Association between CAF-Related signature and immunotherapy prognosis

To investigate the predictive value of the CAF-related signature in immunotherapy, we first analyzed the IMvigor210 cohort. Patients were stratified into high- and low-risk groups based on their CAF risk scores. Survival analysis revealed that patients in the high-risk group exhibited significantly worse overall survival compared to the low-risk group (Fig. 7E**)**. Further analysis demonstrated that CAF risk scores were elevated in patients with progressive disease (PD) compared to those achieving complete response (CR), partial response (PR), or stable disease (SD) (Fig. 7F–G). Among treatment response categories, PD patients consistently showed the highest CAF scores (Fig. 7H). ROC curve analysis confirmed the predictive performance of the CAF signature in this immunotherapy cohort (Fig. 7I).

To validate the association between the CAF signature and immunotherapy response in melanoma, we extended our analysis to two independent melanoma cohorts receiving anti-PD-1 therapy. In the GSE78220 dataset, GSEA indicated significant enrichment of the CAF signature in non-responders (NES = 2.82, adjusted *p* < 0.001; Fig. 7J). A similar trend was observed in the GSE145996 cohort, where the CAF signature also showed enrichment in non-responders (NES = 1.76, adjusted *p* < 0.001, Fig. 7K). Collectively, these findings suggest that a CAF-enriched tumor microenvironment is associated with inferior responses to PD-1 blockade, underscoring the potential of the CAF signature as a biomarker for predicting immunotherapy efficacy.

## Discussion

In our study, by combining scRNA-seq and bulk RNA-seq analyses, we characterized melanoma CAFs and identified 28 prognostic marker genes using LASSO-Cox regression. These genes formed a signature that stratified patients into high- and low-risk groups with significant survival disparities across the discovery and validation cohorts. The CAF-derived risk score was an independent prognostic factor for clinical variables and correlated with CNV and immune infiltration. Additionally, our CAF signature could predict the efficacy of multiple chemotherapy drugs and may serve as a prognostic marker for immunotherapy. Our study also sheds light on the utility of multi-omics integration for developing biologically relevant precision medicine biomarkers.

CAFs are increasingly recognized as pivotal determinants of aggressive tumor behavior and treatment resistance across a spectrum of neoplasms^21–23^. Despite extensive studies on carcinoma models, the functional roles of CAFs in melanoma are not well understood^24^. In this study, we utilized single-cell RNA sequencing (scRNA-seq) to systematically delineate CAF heterogeneity in melanoma. We identified several CAF marker genes consistent with their known roles in biological processes, including extracellular matrix remodeling, cytoskeletal organization, integrin signaling, and cytokine-cytokine receptor interactions^25,26^. The observed enrichment of these pathways substantiates the validity of the CAF signature genes identified in this study. Furthermore, we found that the CAF-related risk score was correlated with lower immune and stromal scores, indicating an immunosuppressive TME dominated by cancer cells. The downregulation of inflammatory pathways and reduction in M1 macrophage populations highlight the immunosuppressive functions of CAFs^27^. Overall, our scRNA-seq analysis provided biological insights into CAFs in melanoma.

We also found the prognostic significance of the CAF signature in predicting survival outcomes in patients with melanoma. Despite the inherent heterogeneity within the TME, the 28-gene CAF signature consistently stratified patients into high- and low-risk cohorts, with differences in survival rates. The predictive accuracy of this risk score surpasses that of conventional clinical variables, underscoring the potential role of TME-centric biomarkers in precision oncology^28^. Our CAF signature has also improved prognostic performance compared to previous signatures derived from bulk tumor profiling^29,30^, possibly due to scRNA-seq-guided gene selection. CAF genes ensure that they are functionally coherent and are intimately connected to the underlying biology of CAFs. Mechanistically, our study revealed a significant correlation between the CAF signature and XIRP2 mutation and CNV. These findings imply that the CAF signature not only reflects transcriptomic disparities but also encapsulates genomic instability within the tumor microenvironment. The mutual interactions between CAFs and cancer cells may coevolve the genomic landscapes in both compartments, leading to enhanced invasiveness and treatment resistance^27,31–33^. Further exploration of these genomic aberrations and their functional implications will provide insights into CAF-modulated tumor progression.

The prognostic CAF risk signature exhibited distinct associations with chemotherapy responsiveness across the molecular subgroups. Notably, imatinib demonstrated enhanced efficacy in patients with high CAF risk scores, which may be attributed to CAFs mediating resistance via the PDGFR signaling pathway. Previous research has indicated that CAFs express receptors such as PDGFR, activating downstream pro-survival signals. Inhibitors targeting PDGFR can reverse CAF-induced resistance, consistent with our findings on imatinib effectiveness^34,35^. Paclitaxel sensitivity in the low-risk group may correlate with the CAFs’ microenvironmental characteristics in this cohort. Axemaker et al.^36^ showed that CAFs can create physical barriers through ECM remodeling, thereby reducing drug penetration. However, the low CAF score group may have a more loosely structured matrix. Overall, while these algorithm-derived predictions underscore stromal cross-talk as a broad mediator of resistance, experimental validation is required to confirm their clinical relevance.

Another finding was the association between the CAF signature and immunotherapy efficacy. Notably, the high-risk group demonstrated diminished survival outcomes after anti-PD1 treatment, and non-responders are associated with CAF signature, indicating that targeting CAFs may improve immunotherapy efficacy. Preclinical studies have shown that CAFs cause resistance to targeted therapies and immune checkpoint inhibitors via ECM remodeling and immunosuppressive cytokine secretion^5,37–40^. CAFs deposit dense collagen and fibronectin networks, creating a barrier against drug delivery and immune cell infiltration, thereby limiting the efficacy of chemotherapy and immunotherapy^41^. This stromal desmoplasia also spatially separates cytotoxic CD8^+^ T cells from tumor nests. Moreover, CAFs secrete TGF-β, IL-6, and Activin A directly suppressing antitumor immunity^42^. Overall, our CAF signature may help predict response and resistance to chemotherapy and immunotherapy.

While many existing signatures predict immunotherapy response based on immune cell infiltration^43^, tumor cells^7,]^ or specific phenotypic gene sets^44^, they often overlook the impact of the stromal compartment. Our CAF-related signature complements these models by capturing the immunosuppressive and physical barrier functions of CAFs, which are key drivers of immune exclusion and therapy resistance. In both the IMvigor210 and melanoma immunotherapy cohorts, the CAF signature could stratify patients with poor response, demonstrating predictive value independent of immune cell activity. Thus, our signature provides a stromal-centric perspective, offering prognostic insights beyond conventional immune-focused models.

Recent advances in spatial transcriptomics (ST) offer a promising avenue for further dissection of CAFs heterogeneity^45,46^. ST preserves the spatial context, enabling the identification of distinct CAF subtypes within their native tumor microenvironment^47^. This spatial resolution is crucial because the functional roles of CAFs, such as immune suppression, matrix remodeling, and therapy resistance, are often influenced by their proximity to tumor cells, vasculature, and immune infiltrates. Incorporating ST can clarify how specific CAF subsets contribute to localized immunosuppressive niches and affect responses to immunotherapy^48,49^. Spatial CAF signatures may also refine current prognostic models by integrating both molecular identity and spatial distribution, paving the way for more precise therapeutic targeting^50^. Future studies leveraging ST are essential to fully elucidate CAF heterogeneity and its clinical implications.

Some limitations should be acknowledged. First, we defined CAFs based on scRNA-seq of a few samples, which may not capture the full spectrum of CAF heterogeneity across different patients and disease stages. Combining additional scRNA-seq datasets could help derive a more comprehensive CAF signature. Second, scRNA-seq has limitations in capturing the spatial heterogeneity and cellular interactions within the TME. Future research could combine spatial transcriptomics with scRNA-seq to provide insights into cellular interactions, which would help verify CAF markers and their roles in tumor progression. Third, although our CAF-based prognostic model demonstrated predictive performance, its generalizability remains to be established in larger, independent cohorts. Likewise, although we observed preliminary associations between the CAF signature and immunotherapy response, these findings are based on limited validation and should be interpreted with caution. Future studies should include prospective clinical studies and additional external datasets to confirm the model’s robustness and explore how CAF-related features influence immunotherapeutic efficacy. Fourth, although preliminary experiments showed that CAF-related genes were highly expressed in melanoma tissues, this did not validate their prognostic value. The prognostic value and functional mechanisms of CAF signature genes also remain to be fully validated.

In summary, by combining scRNA-seq and bulk RNA-seq data, we identified and validated a CAF-related prognostic signature for patients with melanoma. The risk score stratified patients into groups with distinct genomic aberrations, immune landscapes, treatment responses, and survival outcomes. Our study demonstrated the power of multi-omics integration to derive biologically insightful and clinically useful gene signatures. The CAF signature may help develop precision medicine tools for risk stratification and therapeutic decision-making in melanoma management.

## Supplementary Information

Below is the link to the electronic supplementary material.

Supplementary Material 1

## Data Availability

The data and materials used in this study are publicly available in the UCSC Xena database (http://xena.ucsc.edu/) and GEO database(https://www.ncbi.nlm.nih.gov/geo/) with the primary accession code GSE65904, GSE115978, GSE78220 and GSE145996.
