# Supplementary material for: Combining single-cell and bulk RNA sequencing to identify CAF-related signature for prognostic prediction and treatment response in patients with melanoma
Source: Sci Rep. 2025 Aug 8;15:29082. doi: 10.1038/s41598-025-14979-w (PMC12334557; doi:10.1038/s41598-025-14979-w)
Supplement: Supplementary file 1 — Supplementary Material 1 [file 41598_2025_14979_MOESM1_ESM.docx]

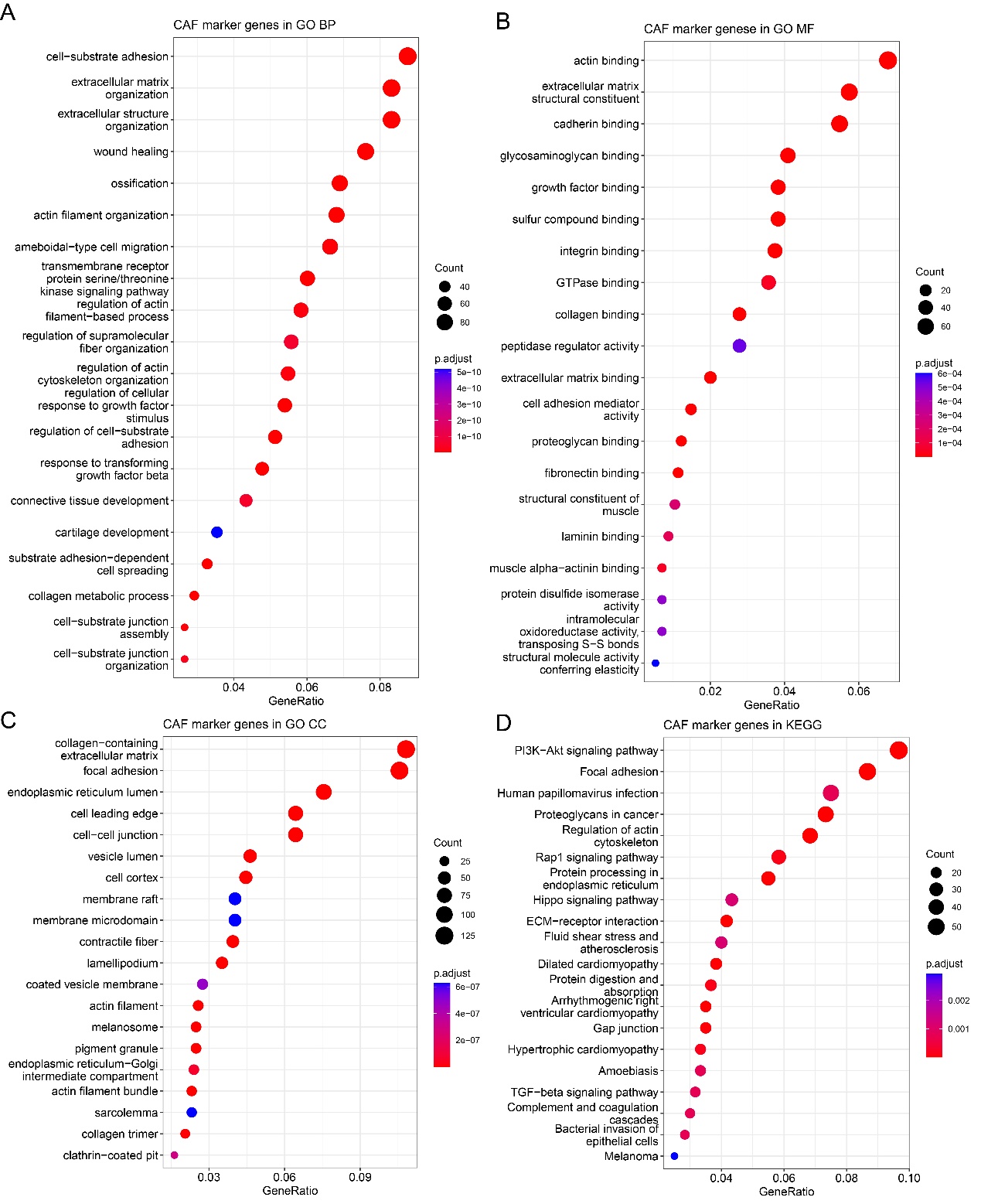


**Supplement Figure 1. GO and KEGG analysis of CAF marker genes.** (A-C) Dot plots showing the GO enrichment of CAF marker genes including biological process, cell components and molecular function. (D) Dot plots showing the KEGG pathways of CAF marker genes.


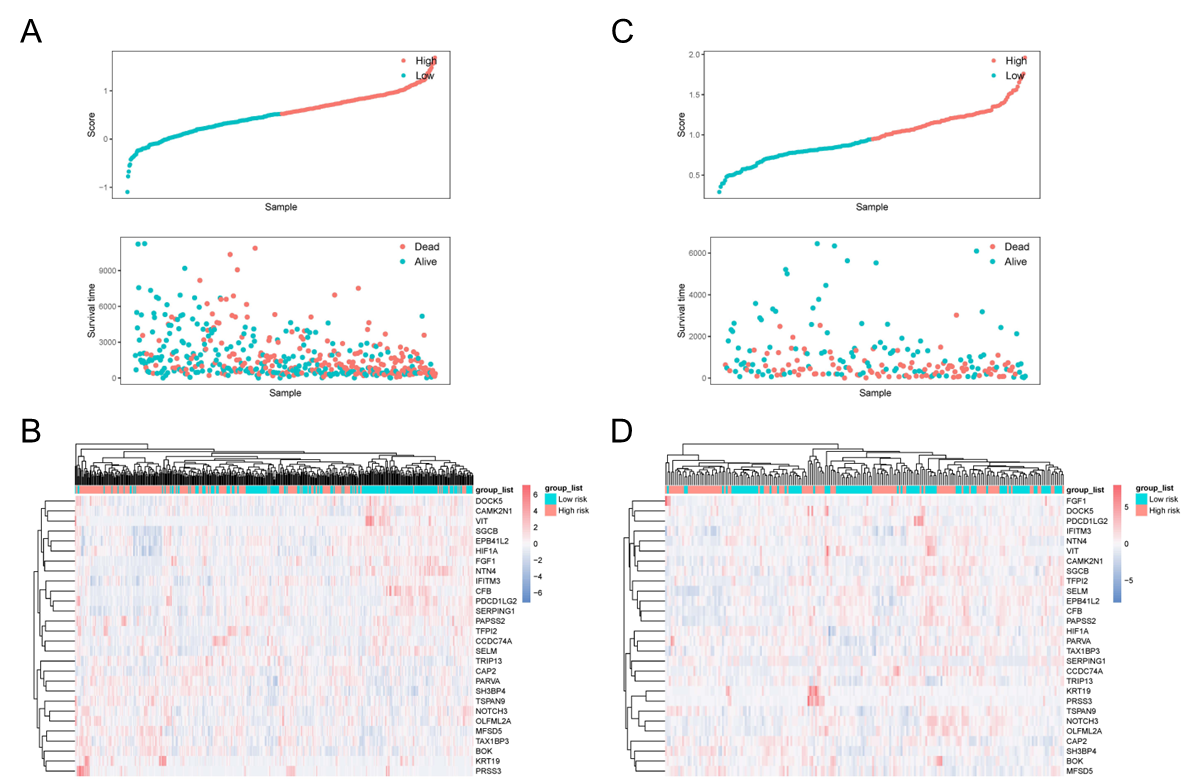


**Supplement Figure 2. CAF related signature in the training dataset and the** **validation dataset**

1. Signature score distribution in patients with melanoma main the training dataset.
2. Heatmap showing the CAF model gene expression in the training dataset.
3. Signature score distribution in patients with melanoma main the validation dataset.
4. Heatmap showing the CAF model gene expression in the validation dataset.


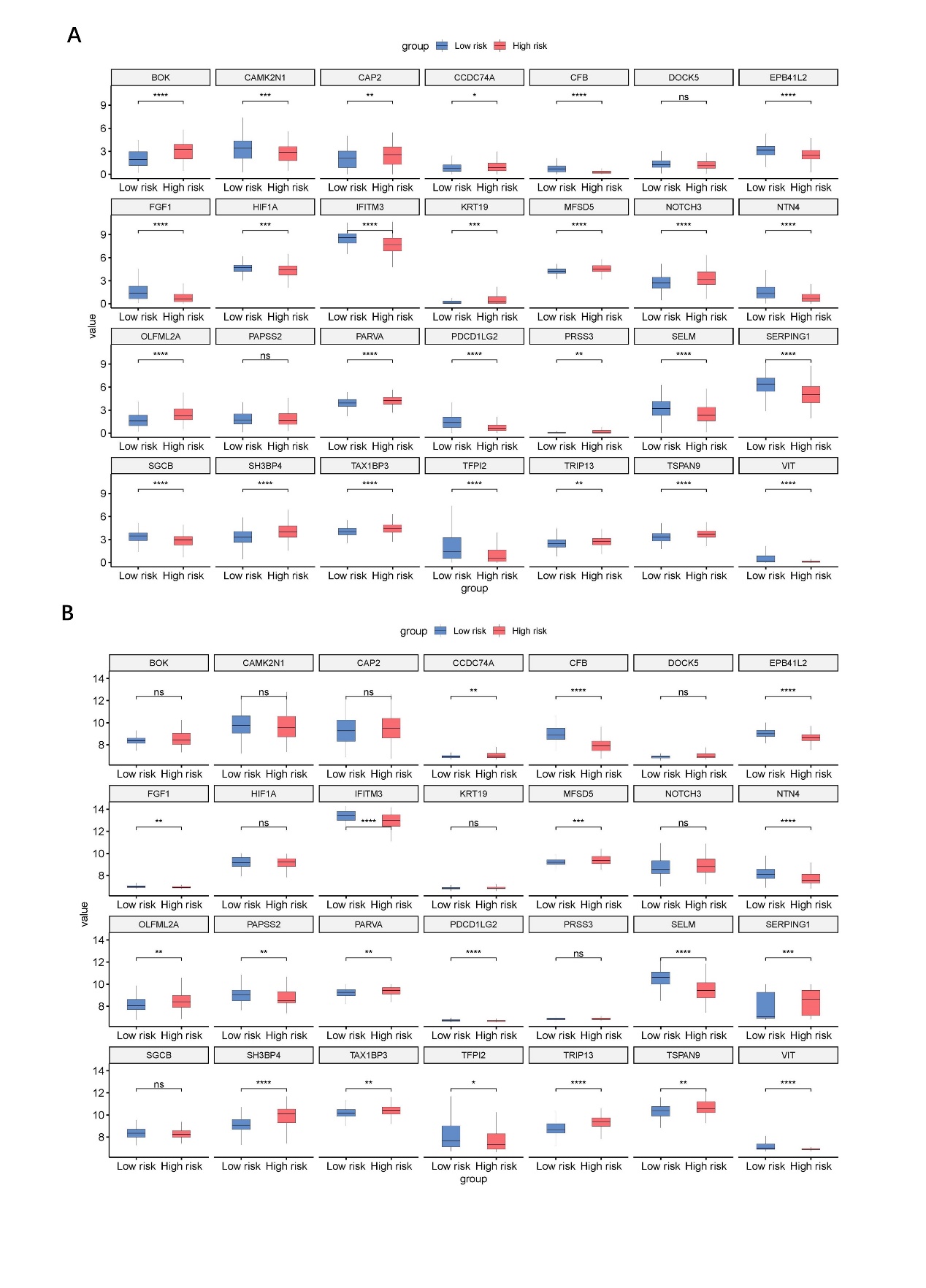


**Supplement Figure 3. Box plots showing** **the CAF model gene expression in the training dataset (A) and test dataset (B).**


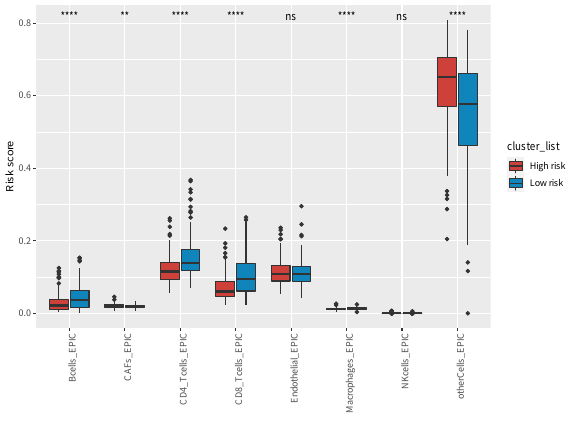


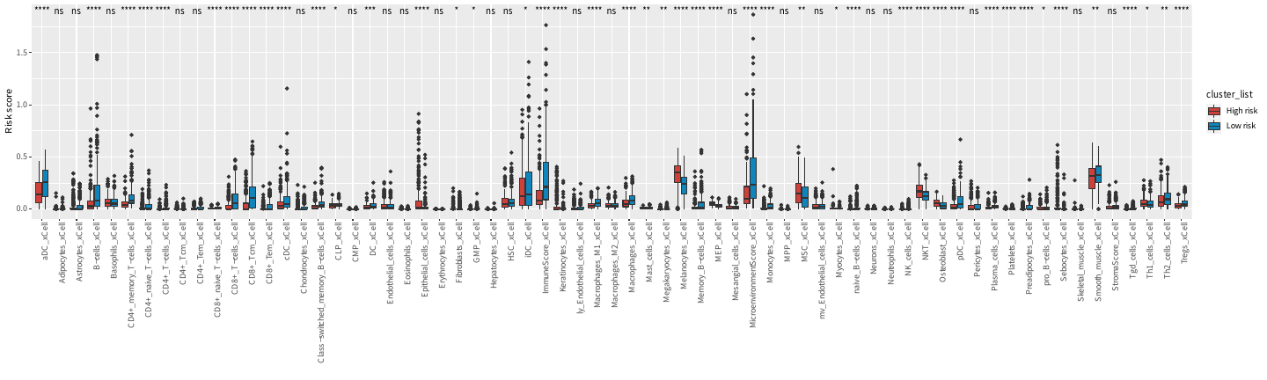


**Supplement Figure 4**. **The percentage of immune cells in different groups estimated by EPIC and XCELL.**

**Supplement Table 1. The demographic characteristics in the training dataset.**

|  | **Alive**  **(N=235)** | **Dead**  **(N=222)** | **Overall**  **(N=222)** |
| --- | --- | --- | --- |
| **Factor (Age)** |  |  |  |
| ＜=60 | 127（54.0%） | 124（55.9%） | 251（54.9%） |
| ＞60 | 108（46.0%） | 98（44.1%） | 206（45.1%） |
| **Radiation therapy** |  |  |  |
| NA | 30(12.8%) | 148(66.7%) | 178(38.9%) |
| NO | 176(74.9%) | 65(29.3%) | 241（52.7%） |
| YES | 29（12.3%） | 9(4.1%) | 38(8.3%) |
| **M Stage** |  |  |  |
| M0 | 207(88.1%) | 200(90.1%) | 407(89.1%) |
| M1 | 13(5.5%) | 11(5.0%) | 24(5.3%) |
| NA | 15(6.4%) | 11(5.0%) | 26(5.7%) |
| **N Stage** |  |  |  |
| N0 | 116(49.4%) | 110(49.5%) | 226(49.5%) |
| N1 | 37(15.7%) | 36(16.2%) | 73(16.0%) |
| N2 | 24(10.2%) | 25（11.3%） | 49（10.7%） |
| N3 | 28（11.9%） | 28（12.6%） | 56（12.3%） |
| NA | 10（4.3%） | 9（4.1%） | 19（4.2%） |
| NX | 20(8.5%) | 14(6.3%) | 34(7.4%) |
| T Stage |  |  |  |
| NA | 29(12.3%) | 28(12.6%) | 57(12.5%) |
| T1 | 25(10.6%) | 16(7.2%) | 41(9.0%) |
| T2 | 37(15.7%) | 40(18.0%) | 77(16.8%) |
| T3 | 40(17.0%) | 50(22.5%) | 90(19.7%) |
| T4 | 80(34.0%) | 68(30.6%) | 148(32.4%) |
| TX | 24(10.2%) | 20(9.0%) | 44(9.6%) |

**Supplement Table 2. The demographic characteristics in the validation dataset.**

|  | **Alive**  **(N=108)** | **Dead**  **(N=102)** | **Overall**  **(N=210)** |
| --- | --- | --- | --- |
| **Age** |  |  |  |
| ＜=60 | 40(37.0%) | 43(42.2%) | 83(39.5%) |
| ＞60 | 67(62.0%) | 59(57.8%) | 126(60.0%) |
| NA | 1(0.9%) | 0(0%) | 1(0.5%) |
| **Gender** |  |  |  |
| Female | 52(48.1%) | 34(33.3%) | 86(41.0%) |
| Male | 56(51.9%) | 68(66.7%) | 124(59.0%) |
| **Tumor tissue** |  |  |  |
| Cutaneous | 18(16.7%) | 3(2.9%) | 21(10.0%) |
| Lymph node | 67(62.0%) | 63(61.8%) | 130(61.9%) |
| NA | 6(5.6%) | 10(9.8%) | 16(7.6%) |
| Subcutaneous | 13(12.0%) | 20(19.6%) | 33(15.7%) |
| Visceral | 4(3.7%) | 6(5.9%) | 10(4.8%) |

| **gene** | **coef** |
| --- | --- |
| MFSD5 | 0.104889 |
| PARVA | 0.085878 |
| PRSS3 | 0.082771 |
| OLFML2A | 0.081982 |
| NOTCH3 | 0.069073 |
| BOK | 0.060082 |
| TSPAN9 | 0.050268 |
| CCDC74A | 0.046397 |
| PAPSS2 | 0.035218 |
| SH3BP4 | 0.032041 |
| TAX1BP3 | 0.017835 |
| KRT19 | 0.017765 |
| CAP2 | 0.016653 |
| TRIP13 | 0.007673 |
| CAMK2N1 | -0.0004 |
| SERPING1 | -0.00191 |
| TFPI2 | -0.0034 |
| HIF1A | -0.00428 |
| EPB41L2 | -0.0146 |
| SGCB | -0.01741 |
| DOCK5 | -0.02139 |
| PDCD1LG2 | -0.0285 |
| SELM | -0.03722 |
| FGF1 | -0.03764 |
| VIT | -0.06772 |
| NTN4 | -0.08842 |
| IFITM3 | -0.10558 |
| CFB | -0.15184 |

**Supplement Table 3. Selected features and their coefficients in the CAF-model.**

**Supplement Table 4. pRRophetic algorithm predicted distinct chemotherapy responses between high- and low-risk CAF groups**

| **Drug** | **P value** | **Sensitivity (AUC values)** |
| --- | --- | --- |
| Bicalutamide | 3.73E-20 | -0.47725 |
| Bryostatin.1 | 3.64E-18 | -0.45124 |
| NSC.87877 | 4.27E-16 | -0.44151 |
| GW.441756 | 2.54E-13 | -0.42241 |
| Imatinib | 1.82E-13 | -0.3838 |
| BI.2536 | 1.02E-14 | -0.37994 |
| Parthenolide | 1.04E-11 | -0.37235 |
| KIN001.135 | 2.91E-11 | -0.34391 |
| OSI.906 | 4.25E-09 | -0.33499 |
| WH.4.023 | 3.20E-09 | -0.3126 |
| GNF.2 | 2.22E-10 | -0.29384 |
| VX.680 | 1.08E-07 | -0.28133 |
| FH535 | 5.38E-07 | -0.2748 |
| Bexarotene | 1.76E-07 | -0.26438 |
| A.443654 | 2.21E-06 | -0.25998 |
| RDEA119 | 1.38E-06 | -0.25388 |
| JW.7.52.1 | 6.13E-07 | -0.25267 |
| MS.275 | 3.74E-06 | -0.24897 |
| Erlotinib | 1.62E-08 | -0.24076 |
| S.Trityl.L.cysteine | 9.34E-06 | -0.23424 |
| JNJ.26854165 | 2.52E-06 | -0.23245 |
| AZD.0530 | 3.86E-06 | -0.22482 |
| KU.55933 | 2.20E-05 | -0.21682 |
| Salubrinal | 0.000125887 | -0.21459 |
| EHT.1864 | 0.000192246 | -0.21456 |
| CMK | 2.46E-06 | -0.20635 |
| Elesclomol | 0.000197396 | -0.1974 |
| NVP.TAE684 | 7.10E-05 | -0.18749 |
| Paclitaxel | 6.07E-05 | -0.18633 |
| GSK269962A | 0.000102414 | -0.18289 |
| PD.0325901 | 0.000266813 | -0.17913 |
| A.770041 | 0.000290571 | -0.17427 |
| BMS.754807 | 0.002346731 | -0.17046 |
| GW843682X | 0.002648204 | -0.17005 |
| Docetaxel | 0.00052775 | -0.16688 |
| FTI.277 | 0.000229495 | -0.16626 |
| CHIR.99021 | 0.036233123 | -0.15785 |
| LFM.A13 | 0.000742355 | -0.14796 |
| WZ.1.84 | 0.000287159 | -0.14509 |
| MG.132 | 0.000517883 | -0.14099 |
| Sorafenib | 0.001343948 | -0.12624 |
| Dasatinib | 0.010296672 | -0.12339 |
| BMS.536924 | 0.004936791 | -0.11859 |
| PHA.665752 | 0.029229628 | -0.11149 |
| AZ628 | 0.026774396 | -0.10157 |
| PLX4720 | 0.030027145 | 0.104106 |
| SL.0101.1 | 0.025418645 | 0.112874 |
| Embelin | 0.045478841 | 0.113829 |
| Pyrimethamine | 0.030912793 | 0.114697 |
| BMS.708163 | 0.047401583 | 0.117148 |
| Doxorubicin | 0.03540999 | 0.123126 |
| Vorinostat | 0.044563776 | 0.136904 |
| Obatoclax.Mesylate | 0.007251967 | 0.139768 |
| AZD7762 | 0.001490235 | 0.161355 |
| AMG.706 | 0.020041309 | 0.16189 |
| CCT007093 | 0.013959652 | 0.167869 |
| PF.02341066 | 0.007713049 | 0.171994 |
| AICAR | 0.000303095 | 0.177267 |
| SB590885 | 4.38E-05 | 0.181281 |
| Gefitinib | 0.000746612 | 0.182162 |
| X681640 | 0.00013288 | 0.197829 |
| CGP.60474 | 0.000495312 | 0.202076 |
| Etoposide | 2.59E-05 | 0.222595 |
| IPA.3 | 1.01E-05 | 0.226466 |
| Bosutinib | 8.51E-05 | 0.228498 |
| NVP.BEZ235 | 0.000524239 | 0.231536 |
| AG.014699 | 0.003126098 | 0.237971 |
| DMOG | 2.56E-05 | 0.246282 |
| Bleomycin | 4.47E-07 | 0.255823 |
| ABT.263 | 5.05E-07 | 0.261046 |
| PAC.1 | 1.38E-07 | 0.267908 |
| ATRA | 7.78E-08 | 0.281896 |
| Cisplatin | 2.49E-08 | 0.297537 |
| BAY.61.3606 | 1.99E-09 | 0.30467 |
| JNK.Inhibitor.VIII | 1.14E-07 | 0.318398 |
| AZD8055 | 3.51E-09 | 0.319432 |
| AZD6482 | 4.29E-09 | 0.329887 |
| Gemcitabine | 1.66E-10 | 0.337569 |
| BX.795 | 6.78E-09 | 0.340511 |
| VX.702 | 1.35E-10 | 0.344169 |
| Mitomycin.C | 3.19E-11 | 0.365196 |
| GDC.0449 | 9.45E-11 | 0.379421 |
| Z.LLNle.CHO | 2.67E-09 | 0.392741 |
| Methotrexate | 1.21E-13 | 0.398596 |
| ZM.447439 | 9.97E-12 | 0.399732 |
| MK.2206 | 1.35E-13 | 0.40656 |
| GDC0941 | 1.95E-12 | 0.40772 |
| Temsirolimus | 1.39E-12 | 0.408645 |
| Lenalidomide | 8.86E-15 | 0.409696 |
| Camptothecin | 3.85E-12 | 0.420993 |
| PD.0332991 | 3.26E-17 | 0.48574 |
| Nilotinib | 1.19E-21 | 0.491028 |
| AZD.2281 | 9.15E-17 | 0.495251 |
| AP.24534 | 1.38E-18 | 0.502968 |
| SB.216763 | 5.61E-20 | 0.512547 |
| ABT.888 | 2.26E-22 | 0.533431 |
